# SAVR and TAVI comparison across the globe based on current regional registry evidence – A meta-analysis of reconstructed time-to-event data^☆^

**DOI:** 10.1016/j.ijcha.2025.101703

**Published:** 2025-05-08

**Authors:** Tulio Caldonazo, Hristo Kirov, Anna Vogel, Angelique Runkel, Murat Mukharyamov, Johannes Fischer, Aryan Dadashzadeh, Torsten Doenst

**Affiliations:** Department of Cardiothoracic Surgery, Friedrich-Schiller-University Jena, Germany

**Keywords:** Surgical aortic valve replacement, Transcatheter aortic valve implantation, Randomized clinical trial, Registry study

## Abstract

**Background:**

There is debate whether surgical aortic valve replacement (SAVR) or transcatheter implantation (TAVI) provide better results for treatment of aortic valve stenosis. While randomized clinical trials (RCTs) are considered to compare the average treatment effect of two methods in a selected patient population, registry data, although biased, reflect every day clinical practice and provide external validation of RCTs. We evaluated the impact of SAVR or TAVI on long-term survival based on local reports from all available regions in the world.

**Methods:**

We systematically searched three databases selecting risk-adjusted registry studies comparing outcomes for SAVR and TAVI with at least five years of follow-up. Reports without all-cause mortality were excluded. One time-to-event curve was reconstructed from survival curves. Cox regression model and sensitivity analysis were performed.

**Results:**

From 10,399 screened studies, 13 met the inclusion criteria with 28,344 patients in the final analysis (follow-up: 5–9 years). In ten studies, survival rates favored SAVR, three showed no difference and none favored TAVI. Hazard ratio (HR) for overall survival was 0.58 (95 %CI: 0.54–0.61, p < 0.01 – favors SAVR). A landmark analysis with a 6-months split showed no significant survival difference in the first 6 months (HR: 0.94, 95 %CI: 0.86–1.02, p = 0.14) and better survival for SAVR compared to TAVI thereafter (HR: 0.43, 95 %CI: 0.40–0.46, p < 0.01). All sensitivity analyses supported this outcome.

**Conclusions:**

This systematic regional registry-type comparison revealed that SAVR is associated with increased long-term survival compared to TAVI, which appears to be independent of the world region in which the study was performed.

## 1 Introduction

There is an ongoing debate, whether surgical aortic valve replacement (SAVR) or transcatheter aortic valve implantation (TAVI) provide better results in the treatment of aortic valve stenosis. A number of randomized controlled trials (RCT) comparing both therapeutic modalities (TAVI and SAVR) have been performed in the last decade, with the aim to provide unbiased clarity and serve as a basis for future treatment recommendations. In parallel, several registry reports on patient outcomes for TAVI compared to SAVR outside of RCTs have been published.

Although RCTs represent the highest quality of evidence and are regarded as the most rigorous way of determining the effect of an intervention on a clinical outcome [1,2], outcomes of RCTs are considered to reflect the average treatment effect often generated in samples from a very selected patient population [3]. In addition, RCTs also suffer from various biases [4]. As a consequence, generalizations of trial findings outside the study population have been questioned [5,6]. In contrast, registry studies are generally burdened with various biases, which usually limits generalizability [7]. However, these data reflect the regional outcomes for a large fraction of the affected patient population in that region and therefore may provide information on the expected regional results [8,9]. This information is not provided by randomized trials. Because both data entities suffer from more or less weaknesses with respect to generalizability, it is important to realize that data from registries serve as external validation of RCT outcomes [1,8,9].

Based on the above considerations, we aimed to evaluate the impact of SAVR or TAVI on long-term survival based on local reports from different regions in the world.

## 2 Methods

Ethical approval of this analysis was not required as no human or animal subjects were involved. This review was registered with the National Institute for Health Research International Registry of Systematic Reviews (PROSPERO, CRD42023427186).

### 2.1 Search strategy

We performed a comprehensive literature search to identify contemporary studies reporting long-term survival between SAVR and TAVI in patients with aortic valve stenosis. Searches were run on May 2023 in the following databases: Ovid MEDLINE, Cochrane Library and ScienceDirect. The complete search strategy is available in Supplementary Table 1.

### 2.2 Study selection

The study selection followed the Preferred Reporting Items for Systematic Reviews and Meta-Analyses (PRISMA) strategy. After de-duplication, records were screened by two independent reviewers (AV and AR). Any discrepancies and disagreements were resolved by a third author (TC). Titles and abstracts were reviewed against pre-defined inclusion and exclusion criteria.

### 2.3 Eligibility criteria

Studies were considered for inclusion if they were written in English and reported direct comparison of endpoints between populations undergoing SAVR and TAVI for aortic valve stenosis. The inclusion also required the availability of reported registry results, with a specific focus on all-cause mortality or survival as the reported endpoint of interest.

Exclusion criteria were non-English publications, studies lacking endpoints of interest, conference abstracts and proceedings, case reports, and non-comparative study designs. Additionally, randomized controlled trials were excluded from consideration. Multinational cohorts or registries without clear data on geographic location were also excluded. The full text was pulled for a second round of eligibility screening. References of the selected articles were also reviewed for relevant studies not captured by the original search. The quality of the included studies was assessed using the Newcastle-Ottawa Scale (Supplementary Table 2). In cases where multiple studies originated from the same center or appeared to involve similar cohorts, we selected only the most recent publication to avoid duplication of patient inclusion. For studies originated from large countries (e.g., United States), it was ensured that they represented different states and regions, thereby minimizing the likelihood of patient duplication.

Two reviewers (AV and JF) independently performed data extraction. Accuracy was verified by a third author (TC). The extracted variables included study characteristics (publication year, country, sample size, study design and risk adjustment strategy) as well as patient demographics (age, sex, mean left ventricular ejection fraction – LVEF, incidence of hypertension – HP, diabetes mellitus – DM, smoking status, prior cerebrovascular event – CVA, myocardial infarction – MI, prior percutaneous coronary intervention – PCI, need of dialysis and chronic obstructive pulmonary disease – COPD.

### 2.4 Primary endpoint

Primary and single endpoint was long-term survival. The included registry studies were risk adjusted reports that compared outcomes for SAVR and TAVI had at least 5 years of follow-up. The geographic location of the selected studies was visualized on a world map.

### 2.5 Statistical analysis

We used reconstructed time-to-event data strategy for the primary endpoint. We used the methods described by Wei et al. to reconstruct individual patient data (IPD) from the Kaplan-Meier curves of all eligible studies for the long-term endpoint [10,11]. Raster and Vector images of the Kaplan–Meier survival curves were pre-processed and digitized, so that the values reflecting to specific timepoints with their corresponding survival/mortality information could be extracted. Where additional information (e.g., number-at-risk tables or total number of events) were available, they were used to further calibrate the accuracy of the time-to-events. Departures from monotonicity were detected using isotonic regression and corrected with a pool-adjacent-violators algorithm [10,11]. To confirm the quality of the timing of failure events captured, we thoroughly checked the consistency with the reported survival or morality data provided in the original publications.

### 2.6 Meta-analysis of reconstructed data

The Kaplan–Meier method was used to calculate the overall long-term survival. The Cox proportional hazards regression model was used to assess between-group differences. For these Cox models, the proportional hazards assumption was verified by plotting scaled Schoenfeld residuals, log–log survival plots, and predicted versus observed survival functions. We plotted survival curves using the Kaplan–Meier product limit method and calculated the Hazard Ratios (HRs) and 95 % CIs of each group. A HR lower than 1 indicated that the survival rates were superior in the SAVR arm.

### 2.7 Sensitivity analyses

As sensitivity analyses, a landmark analysis, a two stage *meta*-analysis and a leave-one-out analysis were performed to check the robustness of the findings. For the two stage *meta*-analysis random-effects DerSimonian-Laird model was performed as previously described [12]. Between-study statistical heterogeneity was assessed with the Cochran Q statistic and by estimating I^2^. High heterogeneity was confirmed with a significance level of p < 0.10 and I^2^ of at least 50 % or more. All statistical analyses were performed using R (version 4.3.1, R Project for Statistical Computing) within RStudio and STATA IC17.0 (StataCorp LLC, College Station, Texas).

## 3 Results

### 3.1 Study characteristics

**Fig. 1:**
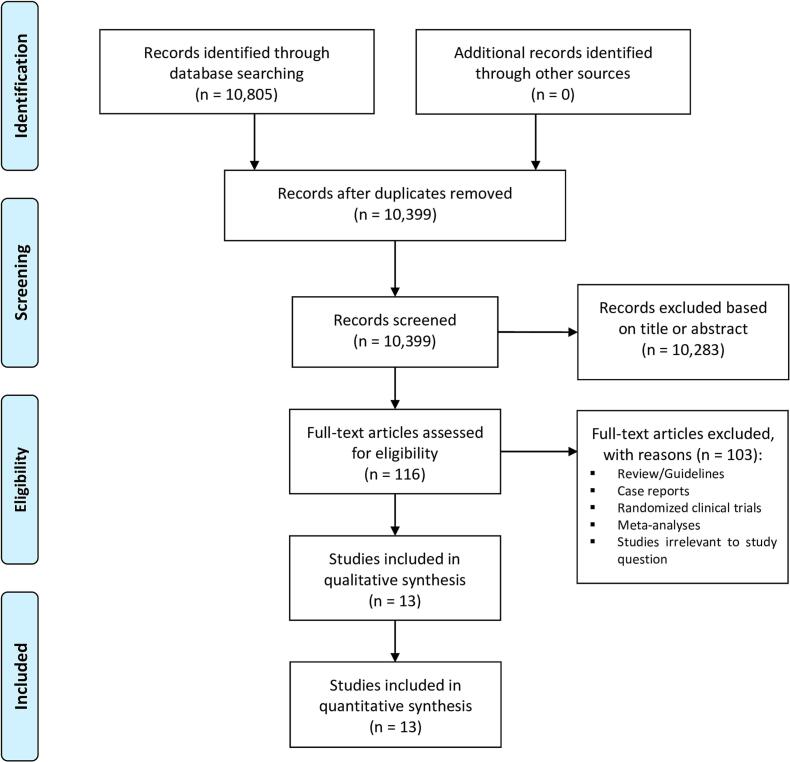
Preferred Reporting Items for Systematic Reviews and Meta-Analyses (PRISMA) flow diagram.

**Fig. 2:**
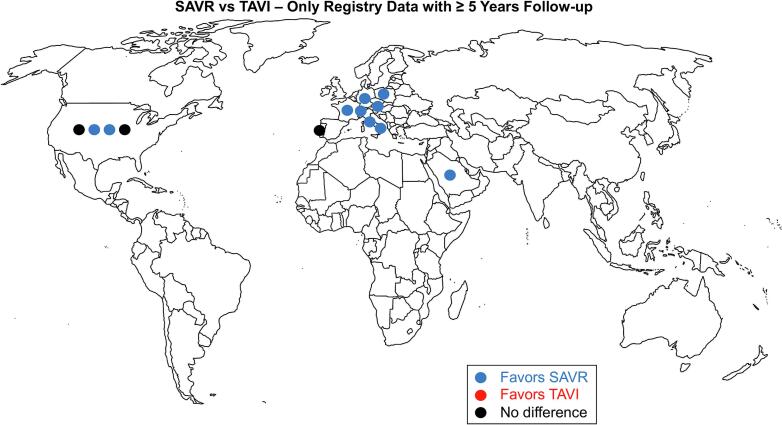
World map showing origin of the included studies. SAVR = surgical aortic valve replacement, TAVI = transcatheter aortic valve implantation.

A total of 10,399 studies were retrieved from the systematic search, of which 13 met the criteria for inclusion in the final analysis. Fig. 1 shows the PRISMA flowchart for study selection. Included studies were published between 2015 and 2023, 8 coming from Europe, 4 from the US and 1 from the Middle East. Fig. 2 shows the geographic location of each registry study.

Table 1 shows the details of the included studies. All studies were based on risk-adjusted populations. A total of 28,344 patients were included in the final analysis.

**Table 1:** Summary of included studies (individual studies’ references in the supplementary material).

Author	Year of Publication	Country	N° of patients	Study Design	Risk adjustment strategy
Arafat	2022	Saudi Arabia	187 70 SAVR, 117 TAVI	Retrospective, Single Center	Multivariable regression
Barbanti	2019	Italy	7,618 5,707 SAVR, 1,911 TAVI	Prospective, Multicenter	Propensity score matching
Beyersdorf	2021	Germany	18,010 9,068 SAVR, 8,942 TAVI	Prospective, Multicenter	Propensity score matching
Bianco	2019	United States	2,379 1,345 SAVR, 1,034 TAVI	Retrospective, Single Center	Multivariable regression
Brizido	2021	Portugal	1,082 682 SAVR, 400 TAVI	Retrospective, Single Center	Propensity score matching
Deharo	2021	France	50,380 28,000 SAVR, 22,380 TAVI	Retrospective, Multicenter	Propensity score matching
Falasa	2022	United States	515 113 SAVR, 402 TAVI	Retrospective, Single Center	Propensity score matching
Kowalkowka	2022	Poland	2,393 1,764 SAVR, 629 TAVI	Retrospective, Multicenter	Propensity score matching
Kramer	2023	United States	164 105 SAVR, 59 TAVI	Prospective, Single Center	Propensity score matching
Mach	2021	Austria	692 604 SAVR, 88 TAVI	Retrospective, Single Center	Propensity score matching
Mack	2015	United States	110 20 SAVR, 90 TAVI	Retrospective, Multicenter	Greedy match algorithm
Santarpino	2021	Italy	1,445 443 SAVR, 1,002 TAVI	Retrospective, Multicenter	Propensity score matching
Vollenbroich	2019	Switzerland	443 107 SAVR, 257 TAVI	Prospective, Single Center	Multivariable regression

### 3.2 Patient characteristics

Supplementary Table 3 summarizes the demographic data of the patient population in each study. Age ranged from 51.0 to 92.0. Percentage of female patients ranged from 3.7 to 100 %; mean LVEF ranged from 30.0 to 57.9; percentage of HP ranged from 44.0 to 89.1 %; percentage of DM ranged from 10.0 to 60.7 %; percentage of current smokers ranged from 3.4 to 73.2 %; percentage of prior CVA ranged from 2.3 to 28.0 %; percentage of prior MI ranged from 4.3 to 46.2 %; percentage of prior PCI ranged from 1.5 to 29.9 %; percentage of prior need of dialysis ranged from 8.3. to 83.0 % and percentage of COPD ranged from 3.9 to 66.0 %.

### 3.3 Overall long-term survival

Overall, 13 Kaplan-Meier curves were processed, digitalized, and reconstructed. Using the previously described methodology, we extracted the IPD from these curves. The entire observation period was up to 9 years.

**Fig. 3:**
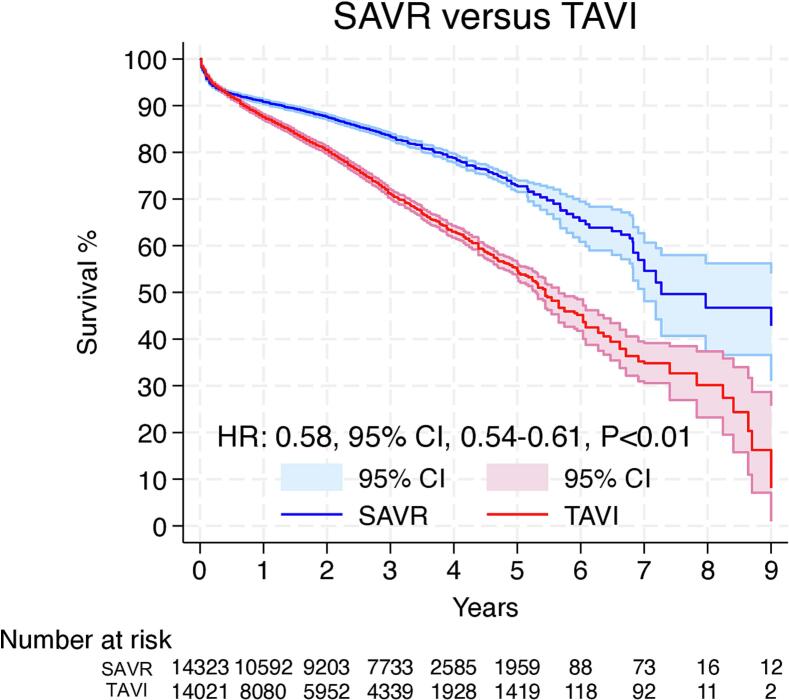
Pooled Kaplan-Meier curves showing long-term survival following SAVR and TAVI. CI = confidence interval, HR: hazard ratio, SAVR = surgical aortic valve replacement, TAVI = transcatheter aortic valve implantation.

Fig. 3 shows the pooled Kaplan-Meier curves for the entire observation period for long-term survival. The patients who underwent SAVR showed better long-term survival compared to the TAVI group (HR: 0.58, 95 % CI, 0.54–0.61, p < 0.01).

### 3.4 Landmark analysis

Violation of the proportional hazards assumption was observed between scaled Schoenfeld residuals and follow-up time, as well as in log–log survival plots, which indicates that the HR is not constant over time (Supplementary Fig. 1). Since we observed that the proportional hazards assumption was violated, we proceeded with landmark analysis, designating 6 months as the landmark timepoint according to the oscillation of HR over time (Supplementary Fig. 2).

**Fig. 4:**
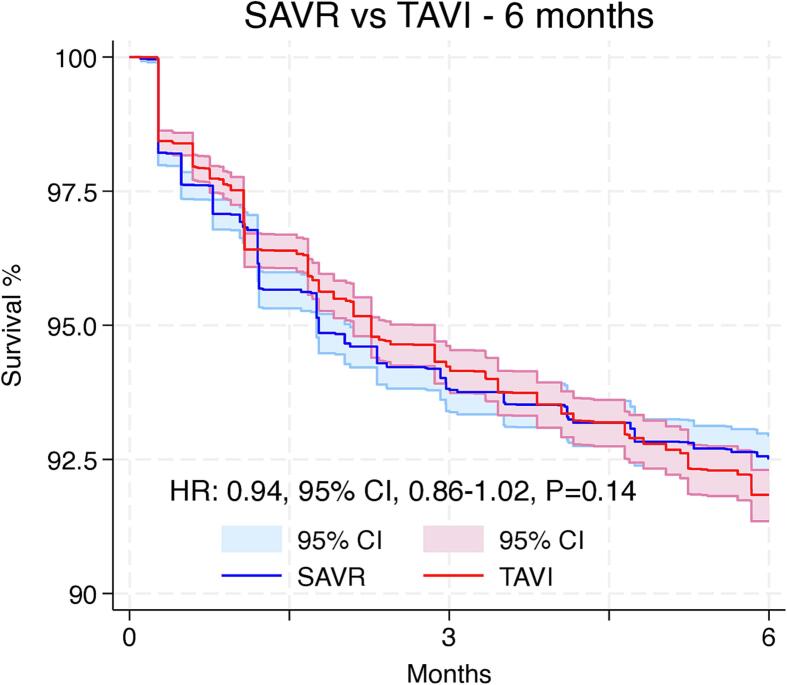

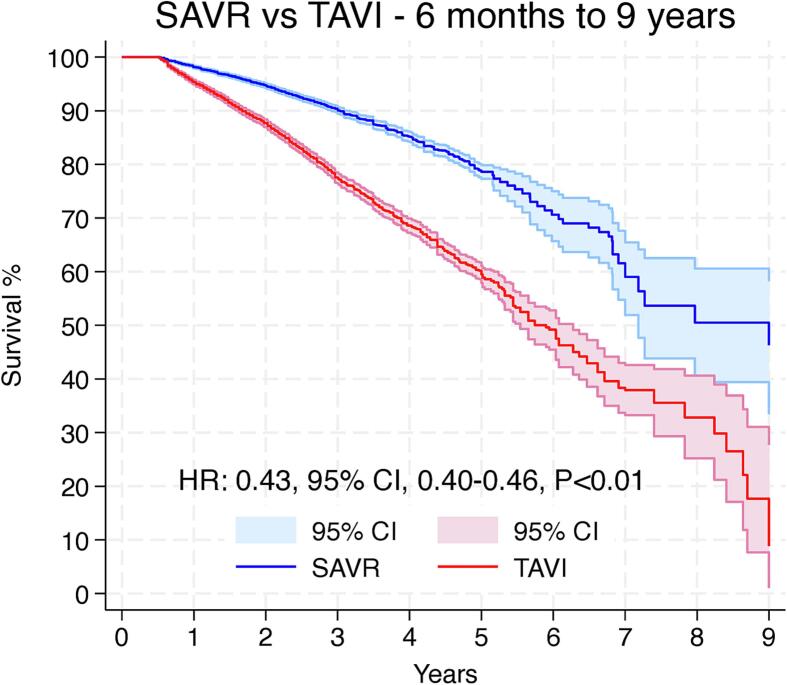
Landmark analysis showing long-term survival following SAVR and TAVI in the initial 6 months (A) and from 6 months to 9 years (B). CI = confidence interval, HR: hazard ratio, SAVR = surgical aortic valve replacement, TAVI = transcatheter aortic valve implantation.

Fig. 4A shows the 6 months survival analysis, which suggested no difference between the groups (HR: 0.94, 95 % CI, 0.86–1.02, p = 0.14). Fig. 4B shows the landmark analysis from 6 months to 9 years, which suggested that compared to patients who underwent TAVI, SAVR was associated with significantly higher survival rates (HR: 0.43, 95 % CI, 0.40–0.46, p < 0.01).

### 3.5 Additional sensitivity analyses

**Fig. 5:**
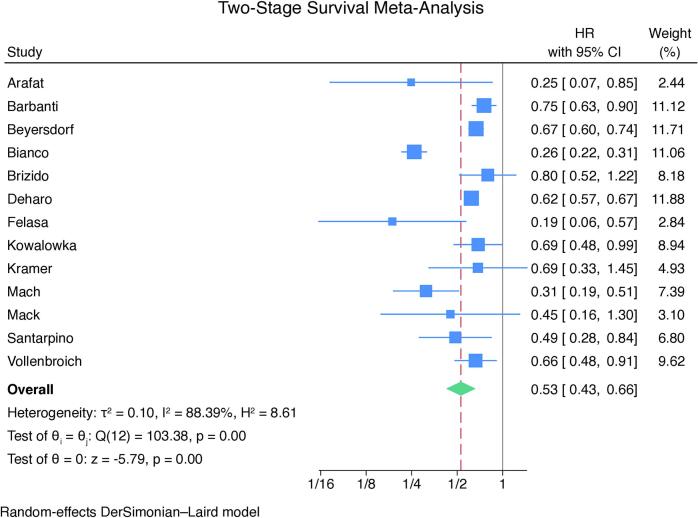

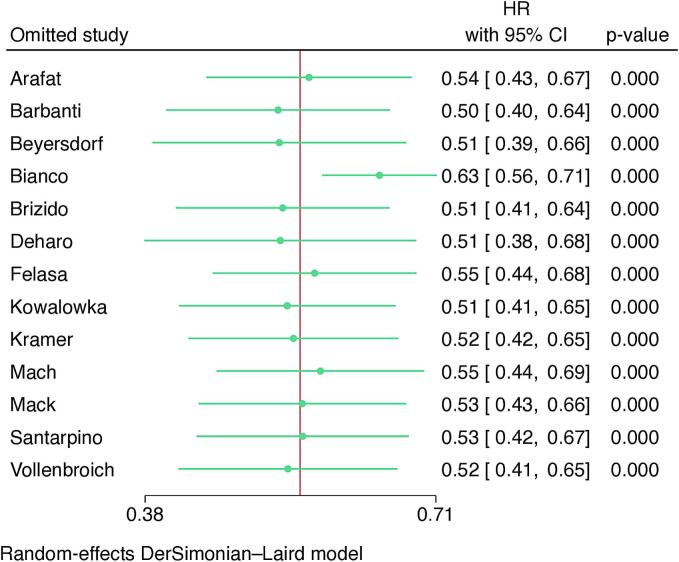
Forest plot (A) and leave-one-out analysis (B) for long-term survival following SAVR and TAVI. CI = confidence interval, HR: hazard ratio, SAVR = surgical aortic valve replacement, TAVI = transcatheter aortic valve implantation.

The same tendency was detected when addressing the individual HR of the studies in the two-stage *meta*-analysis using random-effects model (p < 0.01 – Fig. 5A). No outliers or evidence of publication bias were detected in the leave-one-out analysis (Fig. 5B) and in the funnel plot (Supplementary Fig. 3).

## 4 Discussion

We demonstrate in this systematic regional registry-type comparison that SAVR is associated with increased long-term survival compared to TAVI, which appears to be independent of the region of the world in which the study was performed.

Our results clearly and universally show a long-term survival advantage for SAVR in observational data, which may be contrary to current perception, because most of the individual randomized trials that have been published either show similar outcomes over time [[13], [14], [15], [16]] or early advantages of TAVI [17,18]. Two *meta*-analyses recently systematically assessed the available randomized trials comparing SAVR and TAVI. Barili et al. combined the trials having reported 5 year outcomes until 2020 (mainly high and intermediate risk) and demonstrate slightly better survival for TAVI in the first year and a significant survival advantage for SAVR after 3 years [19]. Pompeu-Sa et al. now demonstrated no difference in survival up to 5 years in comparisons of trials assessing patient with low risk [20]. The just published safety endpoint of the DEDICATE trial then demonstrated superiority of TAVI over SAVR at one year [18]. These data have been and are currently used to support the notion that TAVI is supposed to be the primary treatment modality for the majority of patients receiving biological valves (bicuspid patients may be the exception). As a consequence, two main questions arise. First, how can this significant difference in survival between registry and randomized data be explained, and second – what is the value of observational and randomized data in this context in general.

The observation, that treatment effects in randomized trials are smaller than observed in daily practice (i.e., registry type settings) is not new. In the coronary field, a class IA recommendation to improve survival has recently been issued for “revascularization” of patients with chronic coronary syndrome, although randomized evidence for such an impact has not universally been demonstrated in RCTs [21]. The available data support a survival difference for CABG, specifically in anatomically complex cases, which is also difficult to detect at times [22,23]. The STICH trial, as the most prominent example, had to extend its observation period to 10 years [24] and the most comprehensive *meta*-analysis had to analyze 11 trials with about 11,000 patients to demonstrate 2 % better survival of CABG compared to PCI at 5 years. However, risk-adjusted registry data consistently demonstrate much greater differences consistently [25]. Since, the survival curves in most SAVR-TAVI trials appear to merge or cross with time (in favor of SAVR) and the Barili et al. [19] *meta*-analysis suggests a late survival advantage for SAVR from randomized trials, our findings may not appear that surprising any more.

Another aspect in this context is the fact how the guideline committees deal with the evidence. The new CCS guidelines used a combination of RCT and registry data [26,27] to generate a class IA recommendation for both PCI and CABG [28], which appears reasonable as external validation of RCTs is important.

Although RCTs are considered the gold standard for guiding treatment recommendations, they are not free from bias [4,29] and use highly selected patient populations. Thus, the universal transferability to populations with other characteristics has been questioned [6]. A recent *meta*-analysis by Barili et al. [4] identified significant methodological biases in RCTs comparing TAVI and SAVR. These biases show a consistent pattern, all favoring TAVI, which raises concerns about the internal validity of the studies. Barili et al. [4] point out several key factors contributing to these biases: (I) a substantial proportion of deviations from the assigned treatment, (II) significant loss to follow-up, (III) additional procedures performed during the trials, (IV) an increase in myocardial revascularization interventions and (5 V) systematic selective imbalance. All those biases question the reliability of the RCT results, as they all appear to be skewed in favor of TAVI therapy [4].

In contrast, although heavily burdened with a number of biases, observational data reflect outcomes for a large fraction of the affected patient population and therefore may serve as external validation of RCT results [6,30]. Ideally, registry data confirm RCT findings which consolidates a treatment efficacy and confirms treatment recommendations [30]. When there is a discrepancy, such as in our case, it may be wise to apply caution before making changes in recommendations that dramatically affect patient care. This is specifically relevant in this case, because the unidirectional findings in favor of SAVR in 10 out of 13 analyses (and not a single study showing an advantage for TAVI) questions the equality in longer-term outcomes of the two methods. In Germany, three quarters of patients with relevant aortic stenosis receive TAVI in the meantime [31]. Given that large number, many of them will have life expectancies above 5 years (the observation limit of most randomized trials). Discarding the unidirectional change may translate into many life years lost, if the results presented by us in this analysis are ignored and TAVI indications are even further expanded. A longer- systematic follow up that allows the comparison of TAVI and SAVR beyond 5 years therefore appears critical.

There are pertinent examples from the pharmaceutical industry, where observational data changed the perception of a drug that was introduced into the market with the help of randomized trials. Some drugs had to be withdrawn after successful completion of RCTs and worse results in the post market studies (i.e. cerivastatin). Regulating authorities in this field have recognized the value of observational studies and often oblige sponsors to conduct post-market observational studies (both for medical devices and drugs) after product approval in order to gather additional information about a product's safety, efficacy, or optimal use in the general population [32]. In addition, the new medical device regulation also contains this thought component, where outcome data have to be generated after a device has been approved. Thus, guideline committees may be ill advised by ignoring this validating registry evidence. It is interesting to note in this context, that several of the registry outcomes providing this uni-directional signal for a survival advantage always in favor of SAVR in follow ups to and beyond 5 years [33,34] were already published before the last European guideline recommendations [35], but find no mention in it. A recent position statement on the role of committee members’ conflicts of interest provides an additional perspective [36].

Beyond this point, there is another important aspect worth discussing. It is often argued that the less recent RCTs comparing TAVI and SAVR use transcatheter valves that are outdated and newer generations of valves together with improvement in technique will therefore outperform SAVR. However, this argument is similarly valid for classic surgery, which has also significantly changed (e.g., more minimally-invasive approaches and novel aortic prostheses). Importantly, a significant proportion of the prostheses used in the surgical arms of these trials were valves that have since been withdrawn from the market due to poor performance and high rates of structural valve degeneration (e.g., the Trifecta valve or the Mitroflow). In addition, novel surgical techniques have the potential to achieve superior hemodynamic outcomes implanting prosthesis with proven durability [37]. These considerations raise the question whether current RCT results comparing a single transcatheter valve to a mix of surgical prostheses can truly be used to generalize the individual trial results. The DEDICATE trial may be the first step in the right direction. However, the primary outcome is at five years, but the presented safety endpoint at one year (showing superior results for TAVI over SAVR) seems to be at odds with registry data from the same country [34], where the surgical outcomes seem to be comparable to the surgical arm of DEDICATE but the TAVI registry data are much worse. Thus, an interventionalist bias cannot be excluded from this trial. All these considerations listed above question the internal validity and the generalizability of the conducted RCTs and therefore support the need for external validation by observational data. Our report is therefore an important contribution to this discussion.

Undoubtedly, transcatheter technologies for aortic valve implantation are a valuable tool, providing excellent results for a range of patients and clinical scenarios. However, one interpretation of our findings is that the current implementation of TAVI and the rapid expansion of its indications beyond the patient characteristics tested in RCTs may result in outcomes that are potentially inferior to SAVR for many patients in the long run. Considering the fact that the procedural costs are still higher with TAVI and long-term outcomes have thus far not been better than SAVR, staying with conservative recommendations until more evidence is available, is unlikely going to harm patients. This conclusion is further supported by a recent report applying trial sequential analyses techniques to the available evidence and concludes that the available data do not suffice to safely alter recommendations [38].

Ultimately, this trend is also evident in the context of reintervention, as demonstrated by studies comparing valve-in-valve TAVI (ViV-TAVI) with redo-SAVR. Several authors have commented on this phenomenon, noting that ViV-TAVI could be associated with improved early survival outcomes. However, this initial advantage appears to diminish over time. In fact, pooled data suggest that redo-SAVR may confer better long-term survival [[39], [40], [41], [42]].

## 5 Study strength and limitations

This is the first *meta*-analysis of reconstructed time-to-event data to address this important topic. However, this work has the intrinsic limitations of observational series, including the risk of methodological heterogeneity of the included studies and residual confounders, including a number of different bias already mentioned.

Although all studies included in our analysis employed risk adjustment methods to account for baseline differences between groups, there are some factors which may reflect inherent differences in baseline life expectancy between the groups and cannot be accounted (i.e., patient frailty, anticipated adherence to post-procedural care, heart team deliberations, and individual patient preferences). Moreover, the mere presence of a documented comorbidity may not fully capture its complexity or clinical significance. For instance, standard recordings of conditions like smoking status or diabetes may fail to reflect important factors such as the duration and severity of these conditions or their impact on organ function, which can influence both the short- and long-term outcomes of TAVI or SAVR procedures.

## 6 Conclusion

We demonstrate in this systematic regional registry-type comparison that SAVR is associated with increased long-term survival compared to TAVI, which appears to be independent of the region of the world in which the study was performed.

## Data Availability Statement

The data underlying this article are available in the article and in its online supplementary material.

## CRediT authorship contribution statement

**Tulio Caldonazo:** Writing – review & editing, Writing – original draft, Visualization, Validation, Supervision, Software, Resources, Project administration, Methodology, Investigation, Funding acquisition, Formal analysis, Data curation, Conceptualization. **Hristo Kirov:** Writing – review & editing, Writing – original draft, Visualization, Validation, Supervision, Formal analysis, Data curation, Conceptualization. **Anna Vogel:** Methodology, Formal analysis, Data curation. **Angelique Runkel:** Writing – review & editing, Writing – original draft, Data curation. **Murat Mukharyamov:** Writing – review & editing, Visualization, Validation, Supervision, Resources, Conceptualization. **Johannes Fischer:** Writing – review & editing, Writing – original draft, Conceptualization. **Aryan Dadashzadeh:** Writing – review & editing, Validation. **Torsten Doenst:** Writing – review & editing, Writing – original draft, Validation, Supervision, Resources, Project administration, Conceptualization.

## Funding

TC was funded by the Deutsche Forschungsgemeinschaft (DFG, German Research Foundation) Clinician Scientist Program OrganAge funding number 413668513, by the Deutsche Herzstiftung (DHS, German Heart Foundation) funding number S/03/23 and by the Interdisciplinary Center of Clinical Research of the Medical Faculty Jena.

## Declaration of competing interest

The authors declare that they have no known competing financial interests or personal relationships that could have appeared to influence the work reported in this paper.
