# Supplementary material for: SAVR and TAVI comparison across the globe based on current regional registry evidence – A meta-analysis of reconstructed time-to-event data
Source: Int J Cardiol Heart Vasc. 2025 May 8;59:101703. doi: 10.1016/j.ijcha.2025.101703 (PMC12859432; doi:10.1016/j.ijcha.2025.101703)
Supplement: Supplementary Data 1 [file mmc1.docx]

**Supplementary Material**

**Supplementary Table 1.** Search strategy for Ovid MEDLINE.

**Supplementary Table 2**. Assessment of risk of bias using the Newcastle Ottawa Scale.

**Supplementary Table 3.** Demographics of included patients from the selected studies.

**Supplementary Figure 1.** Test of proportional hazard assumption (A) and in log–log survival plots (B).

**Supplementary Figure 2.** Fluctuation of hazard ratio over time for the entire follow-up.

**Supplementary Figure 3.** Funnel plot for long-term survival.

**Supplementary References**

**Supplementary Table 1.** Search strategy for Ovid MEDLINE.

| ((("surgical procedures, operative"[MeSH Terms] OR ("surgical"[All Fields] AND "procedures"[All Fields] AND "operative"[All Fields]) OR "operative surgical procedures"[All Fields] OR "surgical"[All Fields] OR "surgically"[All Fields] OR "surgicals"[All Fields]) AND ("aortic valve"[MeSH Terms] OR ("aortic"[All Fields] AND "valve"[All Fields]) OR "aortic valve"[All Fields]) AND ("replace"[All Fields] OR "replaceable"[All Fields] OR "replaced"[All Fields] OR "replaces"[All Fields] OR "replacing"[All Fields] OR "replacment"[All Fields] OR "replantation"[MeSH Terms] OR "replantation"[All Fields] OR "replacement"[All Fields] OR "replacements"[All Fields]) AND ("transcatheter aortic valve replacement"[MeSH Terms] OR ("transcatheter"[All Fields] AND "aortic"[All Fields] AND "valve"[All Fields] AND "replacement"[All Fields]) OR "transcatheter aortic valve replacement"[All Fields])) OR ("transcatheter aortic valve replacement"[MeSH Terms] OR ("transcatheter"[All Fields] AND "aortic"[All Fields] AND "valve"[All Fields] AND "replacement"[All Fields]) OR "transcatheter aortic valve replacement"[All Fields] OR ("transcatheter"[All Fields] AND "aortic"[All Fields] AND "valve"[All Fields] AND "implantation"[All Fields]) OR "transcatheter aortic valve implantation"[All Fields])) AND (y_5[Filter]) |
| --- |
| **Translations** |
| **surgical**: "surgical procedures, operative"[MeSH Terms] OR ("surgical"[All Fields] AND "procedures"[All Fields] AND "operative"[All Fields]) OR "operative surgical procedures"[All Fields] OR "surgical"[All Fields] OR "surgically"[All Fields] OR "surgicals"[All Fields]  **aortic** **valve**: "aortic valve"[MeSH Terms] OR ("aortic"[All Fields] AND "valve"[All Fields]) OR "aortic valve"[All Fields]  **replacement**: "replace"[All Fields] OR "replaceable"[All Fields] OR "replaced"[All Fields] OR "replaces"[All Fields] OR "replacing"[All Fields] OR "replacment"[All Fields] OR "replantation"[MeSH Terms] OR "replantation"[All Fields] OR "replacement"[All Fields] OR "replacements"[All Fields]  **transcatheter** **aortic** **valve** **replacement**: "transcatheter aortic valve replacement"[MeSH Terms] OR ("transcatheter"[All Fields] AND "aortic"[All Fields] AND "valve"[All Fields] AND "replacement"[All Fields]) OR "transcatheter aortic valve replacement"[All Fields]  **transcatheter** **aortic** **valve** **implantation**: "transcatheter aortic valve replacement"[MeSH Terms] OR ("transcatheter"[All Fields] AND "aortic"[All Fields] AND "valve"[All Fields] AND "replacement"[All Fields]) OR "transcatheter aortic valve replacement"[All Fields] OR ("transcatheter"[All Fields] AND "aortic"[All Fields] AND "valve"[All Fields] AND "implantation"[All Fields]) OR "transcatheter aortic valve implantation"[All Fields] |

**Supplementary Table 2**. Assessment of risk of bias using the Newcastle Ottawa Scale.

| STUDY | SELECTION | COMPARABILITY | OUTCOME/ EXPOSURE |
| --- | --- | --- | --- |
| Arafat (1) | **** | ** | ** |
| Barbanti (2) | **** | ** | *** |
| Beyersdorf (3) | **** | ** | ** |
| Bianco (4) | **** | ** | *** |
| Brizido (5) | **** | ** | *** |
| Deharo (6) | **** | ** | *** |
| Falasa (7) | **** | ** | ** |
| Kowalowka (8) | **** | ** | *** |
| Kramer (9) | **** | ** | *** |
| Mach (10) | **** | ** | ** |
| Mack (11) | **** | ** | *** |
| Santarpino (12) | **** | ** | ** |
| Vollenbroich (13) | **** | ** | *** |

**Supplementary Table 3.** Demographics of included patients from the selected studies (part 1).

| Study | Age (mean±SD) | | Female (%) | | LVEF (Mean±SD) | | HP (%) | | DM (%) | | Current smoking (%) | |
| --- | --- | --- | --- | --- | --- | --- | --- | --- | --- | --- | --- | --- |
|  | **SAVR** | **TAVI** | **SAVR** | **TAVI** | **SAVR** | **TAVI** | **SAVR** | **TAVI** | **SAVR** | **TAVI** | **SAVR** | **TAVI** |
| Arafat | 51.0 | 75.0 | 12.9 | 27.4 | 32.5 | 30.0 | 54.6 | 75.2 | 28.8 | 60.7 | 15.9 | 3.4 |
| Barbanti | 80.3±5.1 | 80.5±6.2 | 59.5 | 58.9 | 54.2±11.2 | 53.6±11.4 | NR | NR | 25.4 | 24.8 | 11.5 | 10.1 |
| Beyersdorf | 78.0±5.1 | 77.9±6.1 | 51.4 | 51.1 | NR | NR | 89.1 | 87.9 | 34.6 | 33.3 | NR | NR |
| Bianco | 69.1±11.9 | 81.8±7.8 | 41.1 | 50.0 | 56.7±9.9 | 53.3±13.5 | NR | NR | 33.0 | 41.6 | NR | NR |
| Brizido | 79.0±4.0 | 81.0±8.0 | 49.0 | 51.0 | NR | NR | 89.0 | 44.0 | 29.0 | 32.0 | 13.0 | 8.0 |
| Deharo | 79.4±5.8 | 79.4±7.3 | 100 | 100 | NR | NR | 77.9 | 77.5 | 26.5 | 26.4 | 4.4 | 4.2 |
| Falasa | 70.1 | 70.5 | 3.7 | 3.7 | NR | NR | 89.0 | 84.1 | 32.9 | 32.9 | 72.0 | 73.2 |
| Kowalkowa | NR | NR | 56.0 | 62.3 | NR | NR | 63.6 | 64.7 | 27.5 | 33.1 | 39.9 | 38.0 |
| Kramer | 77.0±7.7 | 77.0±9.7 | 52.0 | 59.0 | 50.0±12.0 | 51.0±12.0 | 86.0 | 82.0 | NR | NR | NR | NR |
| Mach | 67.6±7.0 | 68.7±5.5 | 45.8 | 54.2 | 51.7±12.8 | 47.7±11.3 | 73.6 | 86.1 | 12.5 | 11.1 | 12.5 | 25.0 |
| Mack | 92.0±1.9 | 91.8±1.8 | 60.0 | 48.9 | 57.9±11.9 | 53.1±11.7 | 80.0 | 88.9 | 10.0 | 20.0 | NR | NR |
| Santarpino | 80.9±5.1 | 79.1±7.4 | 61.0 | 56.9 | 48.6±7.2 | 49.6±9.9 | 57.5 | 66.2 | 13.9 | 18.6 | NR | NR |
| Vollenbroich | 79.7±5.5 | 82.1±6.2 | 49.5 | 56.0 | 57.0±12.0 | 51.0±14.0 | 79.4 | 78.2 | 19.6 | 24.1 | 15.0 | 16.3 |

DM = Diabetes; HP = Hypertension; LVEF = Left ventricular ejection fraction; NR = Not reported; SAVR = Surgical aortic valve replacement; SD = Standard deviation; TAVI = Transcatheter aortic valve implantation.

**Supplementary Table 3.** Demographics of included patients from the selected studies (part 2).

| Study | Prior CVA (%) | | Prior MI (%) | | Prior PCI (%) | | Need of Dialysis (%) | | COPD (%) | |
| --- | --- | --- | --- | --- | --- | --- | --- | --- | --- | --- |
|  | **TAVI** | **TAVI** | **SAVR** | **TAVI** | **SAVR** | **TAVI** | **SAVR** | **TAVI** | **SAVR** | **TAVI** |
| Arafat | 8.9 | 10.3 | 4.3 | 16.2 | 1.5 | 29.9 | NR | NR | 6.1 | 11.9 |
| Barbanti | NR | NR | 11.5 | 11.1 | 13.1 | 14.5 | NR | NR | 21.7 | 22.3 |
| Beyersdorf | 2.6 | 2.3 | 10.9 | 10.9 | 17.9 | 18.0 | NR | NR | 11.3 | 11.0 |
| Bianco | 5.8 | 13.4 | 17.3 | 38.8 | NR | NR | NR | NR | 19.6 | 32.4 |
| Brizido | 10.0 | 11.0 | NR | NR | NR | NR | NR | NR | 27.0 | 18.0 |
| Deharo | 3.2 | 3.0 | 7.0 | 7.3 | 9.3 | 9.0 | NR | NR | 8.8 | 9.1 |
| Falasa | 17.1 | 19.5 | 13.4 | 12.2 | NR | NR | NR | NR | 20.7 | 22.0 |
| Kowalkowa | 5.1 | 4.6 | 43.0 | 46.2 | NR | NR | 76.6 | 83.0 | 3.9 | 7.0 |
| Kramer | 24.0 | 28.0 | 28.0 | 31.0 | NR | NR | NR | NR | 38.0 | 66.0 |
| Mach | 5.6 | 12.5 | 13.9 | 18.1 | 5.6 | 19.4 | NR | NR | 56.9 | 51.4 |
| Mack | 5.0 | 11.1 | 25.0 | 17.8 | NR | NR | 8.3 | 10.4 | 10.0 | 22.2 |
| Santarpino | NR | NR | NR | NR | NR | NR | 36.0 | 40.1 | 43.6 | 40.1 |
| Vollenbroich | 7.5 | 8.9 | 8.4 | 18.3 | 8.4 | 22.6 | NR | NR | NR | NR |

CVA = cerebrovascular event; MI = Myocardial infarction; NR = Not reported; SAVR = Surgical aortic valve replacement; SD = Standard deviation; PCI = percutaneous coronary intervention; TAVI = Transcatheter aortic valve implantation.

**Supplementary Figure 1.** Test of proportional hazard assumption (A) and in log–log survival plots (B).

A


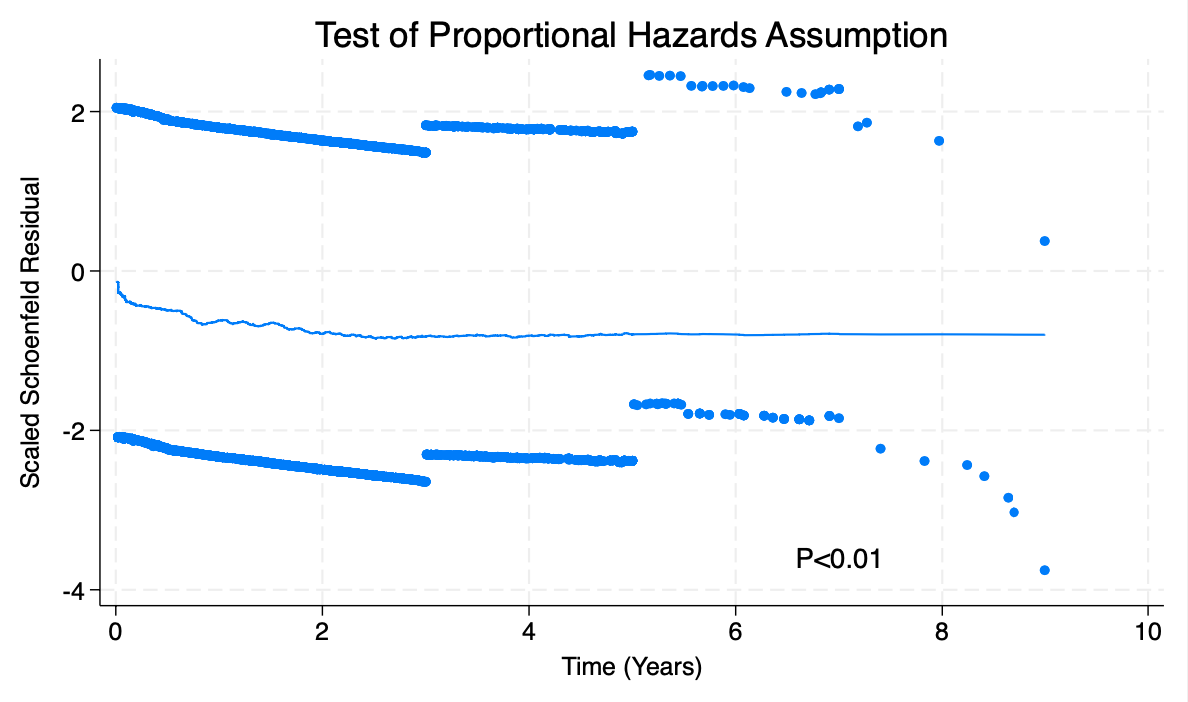


B


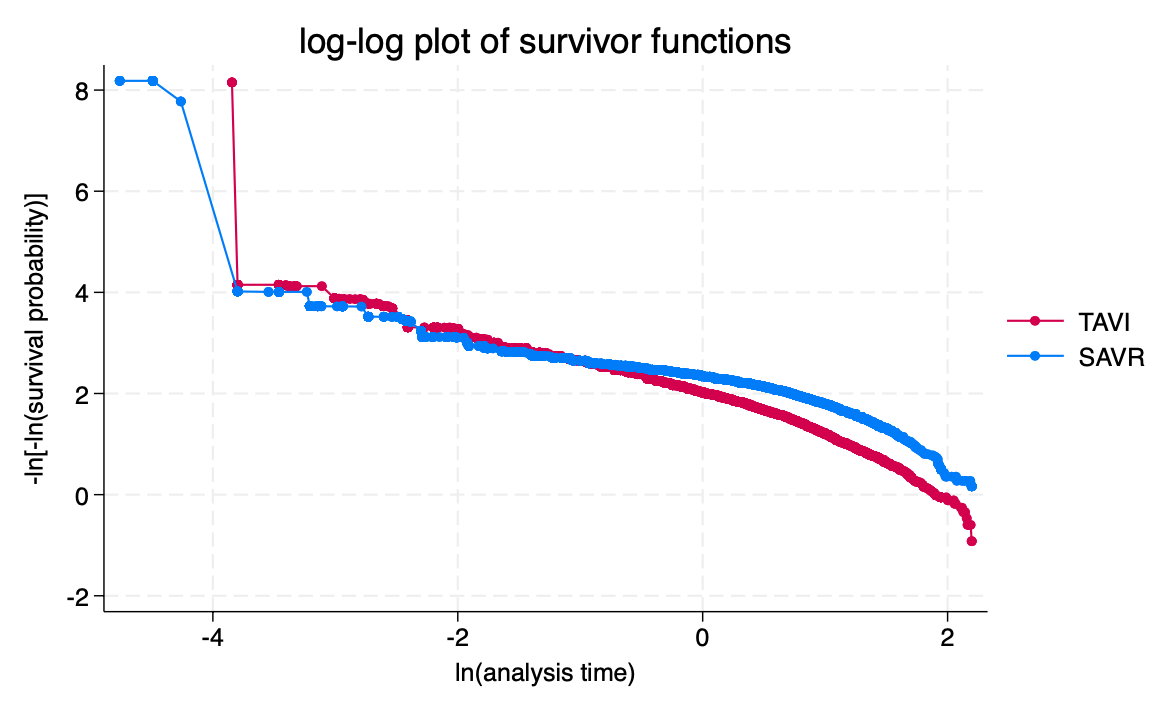


**Supplementary Figure 2.** Fluctuation of hazard ratio over time for the entire follow-up.

**Supplementary Figure 3.** Funnel plot for long-term survival.

**Supplementary References**

1. Arafat AA, Alawami MH, Hassan E et al. Surgical vs Transcatheter Aortic Valve Replacement in Patients With a Low Ejection Fraction. Angiology 2022;74:664-671.

2. Barbanti M, Tamburino C, D'Errigo P et al. Five-Year Outcomes of Transfemoral Transcatheter Aortic Valve Replacement or Surgical Aortic Valve Replacement in a Real World Population. Circ Cardiovasc Interv 2019;12:e007825.

3. Beyersdorf F, Bauer T, Freemantle N et al. Five-year outcome in 18 010 patients from the German Aortic Valve Registry European Journal of Cardio-Thoracic Surgery 2021;60:1139-1146.

4. Bianco V, Kilic A, Gleason TG et al. Long-term Hospital Readmissions After Surgical Vs Transcatheter Aortic Valve Replacement. The Annals of Thoracic Surgery 2019;108:1146-1152.

5. Brízido C, Madeira M, Brito J et al. Surgical versus transcatheter aortic valve replacement in low-risk patients: A long-term propensity score-matched analysis. Catheter Cardiovasc Interv 2021;98:E1033-e1043.

6. Deharo P, Cuisset T, Bisson A et al. Outcomes Following Aortic Stenosis Treatment (Transcatheter vs Surgical Replacement) in Women vs Men (From a Nationwide Analysis). Am J Cardiol 2021;154:67-77.

7. Falasa M, Holmes HR, Neal D et al. Outcome and Cost Comparisons Between Surgical and Transcatheter Aortic Valve Replacements. Innovations 2022;17:482-490.

8. Kowalówka AR, Kowalewski M, Wańha W et al. Surgical and transcatheter aortic valve replacement for severe aortic stenosis in low-risk elective patients: Analysis of the Aortic Valve Replacement in Elective Patients From the Aortic Valve Multicenter Registry. J Thorac Cardiovasc Surg 2022.

9. Kramer B, Vekstein AM, Bishop PD et al. Choosing transcatheter aortic valve replacement in porcelain aorta: outcomes versus surgical replacement. Eur J Cardiothorac Surg 2023;63.

10. Mach M, Poschner T, Hasan W et al. Transcatheter versus Isolated Surgical Aortic Valve Replacement in Young High-Risk Patients: A Propensity Score-Matched Analysis. J Clin Med 2021;10.

11. Mack MC, Szerlip M, Herbert MA et al. Outcomes of Treatment of Nonagenarians With Severe Aortic Stenosis. Ann Thorac Surg 2015;100:74-80.

12. Santarpino G, Lorusso R, Moscarelli M et al. Sutureless versus transcatheter aortic valve replacement: A multicenter analysis of &#x201c;real-world&#x201d; data. Journal of Cardiology 2022;79:121-126.

13. Vollenbroich R, Sakiri E, Roost E et al. Clinical outcomes in high-risk patients with a severe aortic stenosis: a seven-year follow-up analysis. Swiss Med Wkly 2019;149:w20013.
